# Radiotherapy in younger patients with advanced aggressive B-cell lymphoma—long-term results from the phase 3 R-MegaCHOEP trial

**DOI:** 10.1038/s41375-024-02231-9

**Published:** 2024-03-27

**Authors:** Michael Oertel, Marita Ziepert, Fabian Frontzek, Nina Nacke, Bettina Altmann, Maike Nickelsen, Bertram Glass, Viola Poeschel, Christian Ruebe, Georg Lenz, Norbert Schmitz, Hans Theodor Eich

**Affiliations:** 1 Department of Radiation Oncology, University Hospital Muenster, Muenster, Germany; 2 Institute for Medical Informatics, Statistics, and Epidemiology, University of Leipzig, Leipzig, Germany; 3 Centre for Lymphoid Cancer, BC Cancer, Vancouver, BC Canada; 4 Department of Medicine A for Hematology, Oncology, and Pulmonology, University Hospital Muenster, Muenster, Germany; 5 Oncology Lerchenfeld, Hamburg, Germany; 6 Clinic for Hematology, Oncology, Tumor Immunology, and Palliative Care, Helios Klinikum Berlin-Buch, Berlin, Germany; 7 Department of Hematology, Oncology and Rheumatology, Saarland University Medical School, Homburg, Saar Germany; 8 Department of Radiation Oncology, Saarland University Medical School, Homburg, Saar Germany

**Keywords:** Radiotherapy, Chemotherapy

## Abstract

The role of consolidative radiotherapy (RT) for patients with aggressive B-cell lymphoma has not been fully elucidated. The R-MegaCHOEP trial investigated the use of high-dose chemotherapy and rituximab with subsequent autologous stem cell transplantations compared to conventional immunochemotherapy (R-CHOEP) for high-risk patients up to 60 years. The study protocol included RT for patients with bulky (maximum diameter ≥7.5 cm) or extranodal disease. Two-hundred sixty-one patients were analyzed, 120 of whom underwent RT. The most frequently irradiated regions were mediastinum (*n* = 50) and paraaortic (*n* = 27). Median RT dose was 36 Gray in median fractions of 1.8 Gray. Acute toxicities were mostly mild to moderate, with only 24 and 8 grade 3 and 4 toxicities reported during RT. Patients with bulky disease who received RT showed significantly better 10-year EFS, PFS and OS (EFS: 64% vs. 35%; *p* < 0.001; PFS 68% vs. 47%; *p* = 0.003; OS: 72% vs. 59%; *p* = 0.011). There was no significant increase in secondary malignancies with the use of RT. RT administered for consolidation of bulky disease after immunochemotherapy improved the prognosis of young high-risk patients with aggressive B-cell lymphoma and should be considered part of first-line therapy. The *trial was registered with ClinicalTrials.gov, number NCT00129090*.

## Introduction

Diffuse large B-cell lymphoma (DLBCL) is the most frequent lymphoma entity and accounts for roughly one-third of all B-cell lymphomas [1–3]. Immunochemotherapy with rituximab, cyclophosphamide, doxorubicin, vincristine, and prednisone (R-CHOP) is considered the standard first-line therapy achieving long-term survival in approximately 70% of patients [1, 4]. After the incorporation of rituximab, various studies attempted to further improve outcome via integration of novel targeted agents [5, 6]. All these trials failed to meet their endpoints with one exception being the POLARIX study substituting vincristine with the anti-CD79b antibody-drug conjugate polatuzumab vedotin (pola-R-CHP regimen), which resulted in superior progression-free but not overall survival when compared to standard R-CHOP [7]. In younger, high-risk patients with aggressive B-cell lymphoma, the German Lymphoma Alliance (GLA) (formerly German Study Group for High-Grade Lymphomas (DSHNHL)) conducted the R-MegaCHOEP trial investigating dose-intensification of chemotherapeutic agents necessitating sequential high-dose therapy including escalated doses of cyclophosphamide, doxorubicin, and etoposide followed by autologous stem-cell transplantation [8]. This approach, randomized against immunochemotherapy comprising identical drugs at conventional doses (R-CHOEP), failed to meet its endpoint; however, results were among the best ever reported for such high-risk patients [8, 9]. Details of radiotherapy (RT) in patients treated on the R-MegaCHOEP trial have not been presented yet. This remains important, however, as the role of RT as part of first-line therapy for patients with high-risk DLBCL has repeatedly been questioned. Data on RT from a prospective, randomized trial for young-high risk patients with advanced-stage DLBCL have not been published, whereas evidence exist for early-stage, low-risk [10–13] or older patients [14–16] or from pooled retrospective analyses [17], respectively. The current report aims at describing the impact of RT on long-term outcomes of younger patients with high-risk B-cell lymphoma. It also provides a description of toxicities including secondary neoplasia.

## Methods

### Patients and treatment

Between March 3, 2003 and April 7, 2009, the R-MegaCHOEP study enrolled 275 patients with biopsy-proven, untreated, CD20-positive, aggressive B-cell lymphoma. Some patients withdrew their informed consent or had missing data and were not included in the original analysis. Of the remaining 262 patients, RT-information was available for 261 (intention-to-treat population) who were considered in the present analysis (80.5% of patients suffered from diffuse large B-cell lymphoma). Eligibility criteria were age between 18 and 60 years and the presence of two or three risk factors of the age-adjusted International Prognostic Index (aaIPI) (Ann Arbor stage III or IV, elevated lactate dehydrogenase, Eastern Cooperative Oncology Group [ECOG] performance status 2 or 3). A 1:1 randomization between eight cycles of rituximab with cyclophosphamide, doxorubicin, vincristine, etoposide, prednisone in two-week intervals (R-CHOEP) and a high-dose treatment arm comprising escalated doses of cyclophosphamide, etoposide and doxorubicin (R-MegaCHOEP) was utilized. Sequential high-dose therapy necessitated repeated infusions of autologous stem cells after cycles 2, 3, and 4 (for study design see Supplementary Fig. 1). The study protocol stipulated for local RT with 36 Gy to be administered to initial bulky disease and extranodal lesions 3–6 weeks after end of chemotherapy. The original study results and a 10-year follow-up report have been published previously [8, 9]. Here we report details of RT administered within the trial and its impact on study outcomes. The study protocol was approved by local ethics committees and written informed consent was given by all participants. All procedures were carried out in accordance with the declaration of Helsinki.

### Radiotherapy and evaluation

Three to six weeks after the end of immunochemotherapy patients with initial bulky disease (defined as lymphoma masses or conglomerates with diameter ≥ 7.5 cm) and/ or extranodal involvement were to receive consolidative RT if a complete remission (CR), unconfirmed complete remission (CRu), or partial remission (PR) had been achieved (Cheson criteria [18]). A central RT reference panel developed an individual RT plan for each patient. Regarding bulky disease, radiation treatment for infradiaphragmatic, paraaortic, or mesenterial involvement should only be administered after confirmation by the central study office because of putative toxicities. In case of extranodal disease, radiation treatment could be omitted after complete resection of the lymphoma involvement (R0). Liver, bilateral kidney, bilateral adrenal gland, bone marrow, and disseminated extranodal involvements were not irradiated. The recommended dose for radiation treatment was 36 Gy in fractions of 1.8 Gy-2.0 Gy 5 times a week as involved-field radiotherapy independent from the response to immunochemotherapy. Treating physicians at the participating study centers decided if patients should be referred for RT at one of the designated participating institutions. After RT, the final remission status of each individual patient was determined via a CT-scan. For the current analysis, all case report forms (CRF) including all follow-up information collected over time were reviewed again and final decisions were made if RT had been administered as per protocol (radiation dose and field). In particular, two radiotherapists not involved in study design and conduct (HTE and MO) decided, which patients experienced protocol violations (patients with initial bulky or extranodal disease not irradiated and patients without indication as per protocol but receiving RT without documented relapse or progression).

### Statistical analysis

Details of statistical analyses have been previously reported [8, 9]. The primary endpoint of the MegaCHOEP study was event-free survival (defined as time from randomization to disease progression, start of salvage treatment, additional, unplanned treatment, relapse, or death from any cause). Secondary survival endpoints were progression-free survival (defined as time from randomization to progression, relapse, or death from any cause) and overall survival (defined as time from randomization to death from any cause). Continuous variables were summarized by the median values and range, whereas categorical variables are presented as absolute numbers and relative frequencies. Pearson’s chi-square/ Fisher exact tests were used to test for correlations between categorical variables. Event-free survival (EFS), progression-free survival (PFS), and overall survival (OS) were estimated according to Kaplan-Meier, differences between groups were compared by log-rank tests. Kaplan-Meier estimates at 10 years, with 95% confidence intervals (CI), were calculated. Statistical significance was defined as a *p* value of <= 0.05. All statistical analyses were carried out using SPSS version 28 (IBM, Armonk, NY, USA) and Microsoft Excel (Microsoft Corporation, Redmond, Washington, USA).

## Results

### Patient characteristics

**Fig. 1:**
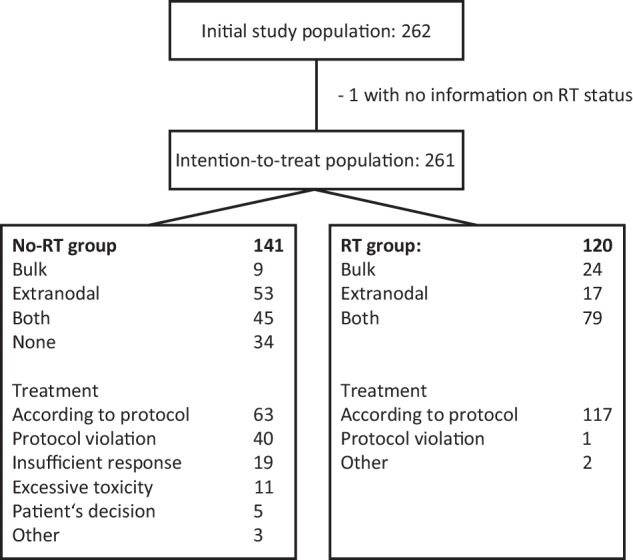
Consort diagram. Consort diagram displaying patient numbers included within the study with or without radiation treatment and numbers for extranodal and bulky disease.

Major patient characteristics and a diagram describing patient flow with regard to RT are shown in Table 1 and Fig. 1. Median follow-up of all patients was 9.3 years.

**Table 1:** Patient characteristics for the whole study population and patients with bulky disease in comparison between the group undergoing radiotherapy (RT) and the group without RT (no-RT).

	All patients		Patients with bulky disease	
	No-RT (*n* = 141)	RT (*n* = 120)	No-RT (*n* = 54)	RT (*n* = 103)
Sex				
Male	85 (60%)	79 (66%)	30 (56%)	69 (67%)
Female	56 (40%)	41 (34%)	24 (44%)	34 (33%)
Age, years	49 (18–60)	45 (18–60)	48 (24–60)	44 (18–60)
Lactate dehydrogenase level				
Elevated more than normal	138 (98%)	116 (97%)	53 (98%)	101 (98%)
Ann Arbor stage				
III or IV	140 (99%)	112 (93%)	53 (98%)	95 (92%)
ECOG performance status				
0–1	97 (69%)	78 (65%)	30 (56%)	69 (67%)
>1	44 (31%)	42 (35%)	24 (44%)	34 (33%)
B-symptoms*				
Yes	86 (61%)	66 (55%)	37 (68%)	59 (57%)
No	54 (39%)	53 (45%)	17 (32%)	44 (43%)
Bone marrow involvment				
Yes	13 (9%)	13 (11%)	7 (13%)	9 (9%)
No	128 (91%)	107 (89%)	47 (87%)	94 (91%)
Age-adjusted International Prognostic Index				
2	101 (72%)	90 (75%)	32 (59%)	79 (77%)
3	40 (28%)	30 (25%)	22 (41%)	24 (23%)
Bulky disease				
Yes	54 (38%)	103 (86%)	–	–
No	87 (62%)	17 (14%)	–	–
Histology				
Not Reviewed	7 (5%)	4 (3%)	2 (4%)	3 (3%)
Reviewed	134 (95%)	116 (97%)	52 (96%)	100 (97%)
DLBCL	102 (76%)	99 (85%)	38 (73%)	83 (83%)
Follicular lymphoma (grade III)	5 (4%)	5 (4%)	2 (4%)	5 (5%)
Follicular lymphoma and DLBCL	4 (3%)	2 (2%)	1 (2%)	2 (2%)
Burkitt’s lymphoma	–	1 (1%)	–	1 (1%)
Burkitt-like lymphoma	1 (1%)	1 (1%)	1 (2%)	1 (1%)
Blastic mantle-cell lymphoma	2 (2%)	–	2 (4%)	–
Aggressive marginal-zone lymphoma	3 (2%)	–	1 (2%)	–
Unclassified B-cell lymphoma	12 (9%)	5 (4%)	5 (10%)	5 (5%)
No aggressive B-cell lymphoma	4 (3%)	1 (1%)	1 (2%)	1 (1%)
Technically insufficient material	1 (1%)	2 (2%)	1 (2%)	2 (2%)

*DLBCL* diffuse large b-cell lymphoma, *ECOG* eastern cooperative oncology group *Information on B-symptoms was missing in one patient in every cohort, percentages are adjusted to available data.

### Radiotherapy

One-hundred-twenty of 261 patients (46%) were irradiated, 52 of whom had reached a complete (CR) or unconfirmed complete remission (CRu) according to the Cheson criteria [18] at the end of immunochemotherapy, 50 patients were irradiated for PR or less than PR at the end of immunochemotherapy (18 patients with unknown response after chemotherapy; Supplementary Table 1). Patients with RT were predominantly male (66%), almost uniformly showed an elevated LDH (97%), about two-thirds had ECOG scores of 0-1 (65%) (Table 1). Compared to the no-RT group, there were more patients in stage I/II in the RT group (1% vs. 7%, for the no-RT vs. RT-group, respectively). These early-stage patients uniformly had bulky disease.

RT was administered to 49.6% of patients in the R-CHOEP arm and 42.4% in the Mega-CHOEP arm (*p* = 0.244). Twenty-six (50%) and 26 (50%) patients were irradiated after reaching a CR/CRu, 32 (64%) and 18 (36%) patients received RT for insufficient response (PR, SD) in the R-CHOEP and the R-MegaCHOEP arm, respectively. 47% and 43% of patients with aaIPI 2 and 3 were irradiated with no difference in aaIPI between the irradiated and the no-RT group (*p* = 0.540).

The regions most frequently irradiated were mediastinum (50), paraaortic (27), and mesenteric (15) regions, followed by left iliac lymph nodes (12) and bone (12). Median RT dose was 36 Gy (5.4 Gy- 46 Gy) with single doses of 1.8 Gy (1.5 Gy–2 Gy).

Extranodal involvement occurred most frequently in the pleura (29), pericardium (26), spleen (24), lungs (23) and skeleton (13 below and 5 above the diaphragm, 4 both). Bulky disease at diagnosis was present in 103 of 120 irradiated patients in comparison to 54 of 141 patients in the non-irradiated group (Table 1). In the RT-group, bulky disease had a maximum width of 7.5–20.0 cm (median: 11.0 cm, lower quartile: 9.0 cm, upper quartile: 14.2 cm). Bulky disease was located predominantly in the mediastinal (44), paraaortic (17) and mesenteric (13) region. A second and third bulk was found in 21 and 5 patients, respectively. The main location for a second bulk was the paraaortic region (7), there was no predominant pattern for a third bulk (5 different regions). Of the 103 patients irradiated with bulky disease, 101 (98%) were treated according to protocol (2 for other reasons). Characteristics of all patients with bulky disease both in the RT and the no-RT group are displayed in Table 1.

### Outcomes

#### Outcomes—intention-to-treat (ITT) population

**Fig. 2:**
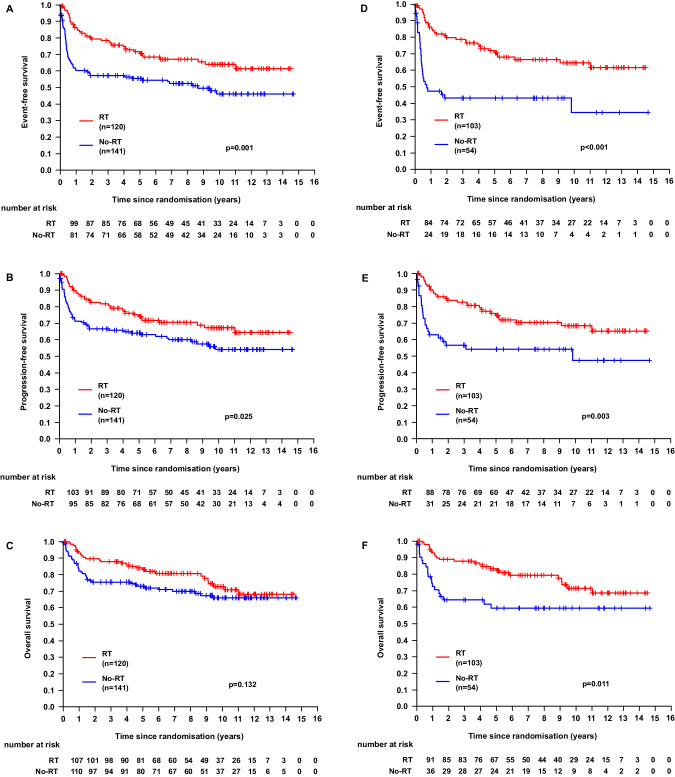
Survival curves for the intention-to-treat population and patients with bulky disease. **A–C** Survival curves for the whole patient collective. Event-free survival (**A**), progression-free survival (**B**) and overall survival (**C**) for the patients undergoing RT (red) in comparison to those without RT (blue). Outcomes were compared after a 10-year follow-up and compared using a log-rank test. **D–F** Survival curves for patients with bulky disease. Event-free survival (**D**), progression-free survival (**E**) and overall survival (**F**) for the patients undergoing RT (red) in comparison to those without RT (blue). Outcomes were compared after a 10-year follow-up and compared using a log-rank test.

For the ITT population, 10-year EFS was 54% (95%-confidence interval (95%-CI): 48–61%), 10-year PFS was 60% (95%-CI: 53–67%) and 10-year OS was 69% (95%-CI: 63–76%). There were significant differences for patients treated with RT in comparison to the group without RT regarding EFS (64%, 95%-CI: 54–74% vs. 46%, 95%-CI: 36–56%; *p* = 0.001) and PFS (67%, 95%-CI: 58–77% vs. 54% 95%-CI: 44–64%; *p* = 0.025) but not OS (73%, 95%-CI: 63–82% vs. 66%, 95%-CI: 57–75%; *p* = 0.132; Fig. 2A–C). Limiting the analysis to patients with a CR/CRu after completion of immunochemotherapy, patients with RT did not differ significantly compared to the non-irradiated group regarding EFS (*p* = 0.798), PFS (*p* = 0.692) and OS (*p* = 0.367). For patients with DLBCL, there was a significant improvement in 10-year EFS with RT (*p* = 0.019). As there has been no randomization concerning RT, we further compared the outcomes in the two treatment arms within the RT-group. There were no differences in EFS (*p* = 0.816), PFS (*p* = 0.555) or OS (*p* = 0.190).

#### Outcome—extranodal and bulky disease

Considering extranodal lesions only, there was a difference in EFS (10-year EFS: 62%, 95%-CI: 51–72% for patients with RT vs. 51%, 95%-CI: 40–62% for patients without RT; *p* = 0.017) but not in PFS or OS (PFS: *p* = 0.068; OS: *p* = 0.305). In contrast, we observed significant differences in all survival endpoints for patients with bulky disease. Ten-year EFS (64%, 95%-CI: 54–75% vs. 35%, 95%-CI: 16–53%; *p* < 0.001), 10-year PFS (68%, 95%-CI: 58–78% vs. 47%, 95%-CI: 30–65%; *p* = 0.003) and most importantly 10-year OS (72%, 95%-CI: 61–82% vs. 59%, 95%-CI: 45–73%; *p* = 0.011; Fig. 2D–F) were all significantly better for patients with RT.

**Fig. 3:**
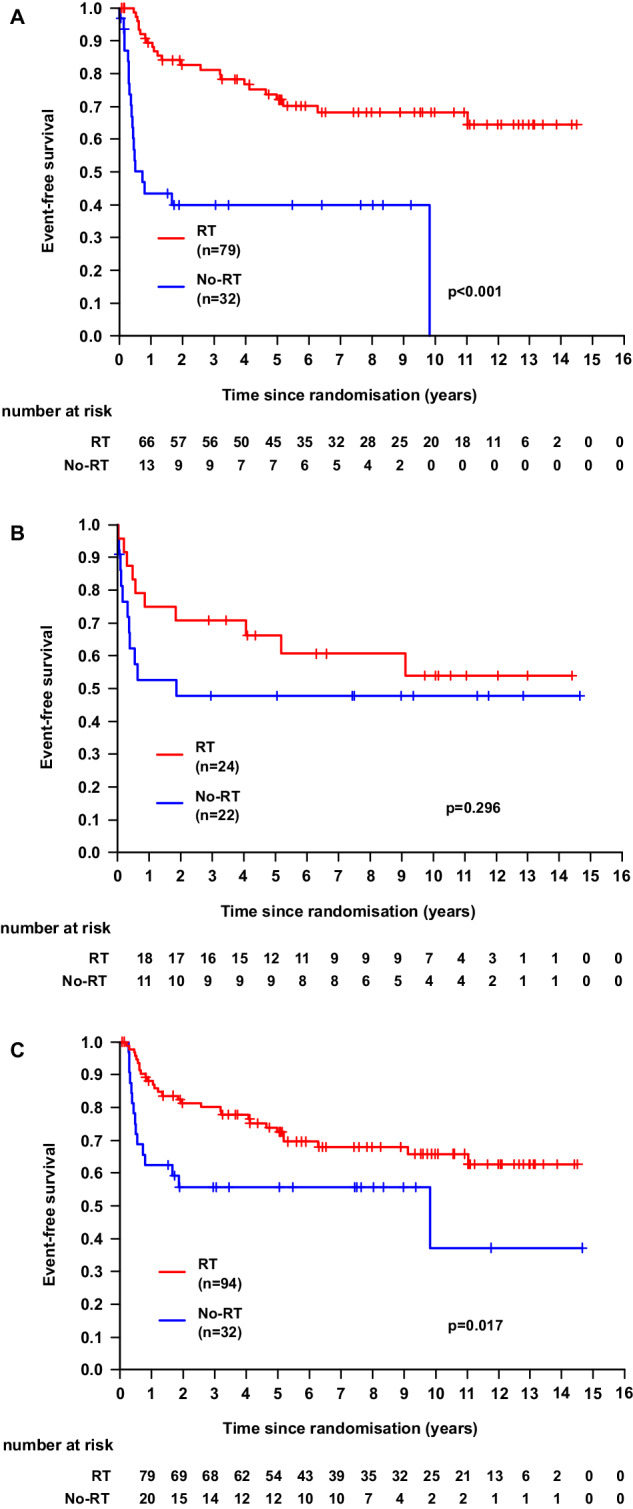
Survival curves for subgroups of patients with bulky disease. Event-free survival (EFS) for patients undergoing RT (red) in comparison to those without RT (blue). Subgroups of patients with an age-adjusted international prognostic index of 2 (**A**) or 3 (**B**) or with completion of chemotherapy as intended (**C**), respectively. Outcomes were compared after a 10-year follow-up and compared using a log-rank test.

As in the group of patients with bulky disease, there was an imbalance regarding IPI categories between irradiated and non-irradiated patients (see Table 1), further analyses were done. There was a significant improvement in 10-year EFS for patients with bulky disease undergoing RT with an aaIPI of 2 (68%, 95%-CI: 57–79% vs. not reached; *p* < 0.001), but not with an aaIPI of 3 (*p* = 0.296) (Fig. 3A, B). Similar results were observed for PFS and OS when patients with aaIPI 2 and 3 were analyzed separately (data not shown). To exclude confounding by early termination of chemotherapy, an analysis of patients with bulky disease who completed immunochemotherapy as intended was done confirming a significant improvement in EFS with RT (10-year EFS: 66%, 95%-CI: 51–74% vs. 37%, 95%-CI: 5–69%; *p* = 0.017; Fig. 3C). However, for patients with bulky disease reaching a CR/CRu after systemic therapy, no difference in EFS was found with the use of RT (*p* = 0.693). Limiting the analysis to patients with DLBCL did not alter the impact of RT on outcome parameters (EFS: *p* < 0.001; PFS: *p* = 0.016; OS: *p* = 0.026). Also in the bulky subgroup, no differences in outcomes between the two treatment arms were found for the irradiated patients (EFS: *p* = 0.952; PFS: *p* = 0.699; OS: *p* = 0.121).

#### Protocol violations

Only one patient not qualifying underwent RT; in contrast, forty patients assigned to RT as per protocol did not receive it as a protocol violation. Twenty-one of these patients had an extranodal involvement being amenable to RT and 19 revealed a bulky disease (with no prior R0-resection). Thirty-one of forty patients (77.5%) had at least one localization challenging to irradiate (gastrointestinal tract, lungs, pancreas, pleura, reproductive organs, or the central nervous system). Other reasons for radiation not to be carried out were insufficient response to immunochemotherapy (19 patients), excessive toxicity (11 patients), patients wish (5 patients) or other (3 patients; Fig. 1).

To estimate the impact of patients who did not receive RT by protocol violation, we compared the patients undergoing RT with the non-irradiated group after exclusion of these 40 patients. The results obtained after exclusion of patients with protocol violations were comparable overall (EFS: *p* < 0.001; PFS: 0.003; OS: 0.043) and in the subgroup with bulky disease (EFS: *p* < 0.001; PFS: *p* < 0.001; OS: *p* < 0.001).

#### Toxicities

Toxicities attributed to RT were mostly mild to moderate with 24 grade 3 and 8 grade 4 toxicities (Table 2). Most toxicities, especially of grades 3 or 4 were hematologic (19/24 and 8/8, respectively), with leukocytopenia being most frequently reported (14 and 6 cases, respectively). Typical RT-associated acute side effects like skin toxicities or dysphagia were limited to grade 1 and 2 (28 and 3 or 23 and 6 cases, respectively). The previously reported toxicities of immunochemotherapy and RT included grade 3–4 mucositis in 8.3% (R-CHOEP) and 64.8% (R-MegaCHOEP) of patients, respectively [8]. All patients in the high-dose therapy arm had grade 4 leukopenia and grade 3–4 thrombocytopenia in comparison to 58.5% leukocytopenia and 33.8% grade 3–4 thrombocytopenia in the R-CHOEP arm [8].

**Table 2:** Overview on acute toxicities documented during radiotherapy.

Toxicity category	Grade 1	Grade 2	Grade 3	Grade 4
Hematological toxicities				
White blood cells decreased	13	14	14	6
Anemia	13	5	3	0
Platelets count decreased	7	9	2	2
Non-hematological toxicities				
Skin	28	3	0	0
Dysphagia	23	6	0	0
Diarrhea	7	2	0	0
Nausea	6	3	0	0
Mucositis	5	5	1	0
Vomiting	4	1	0	0
Constipation	1	0	0	0
Nervous system disorders	4	2	0	0
Larynx	3	1	0	0
Salivary Gland	2	0	0	0
Psychiatric disorders	2	1	0	0
Alopecia	1	1	2	0
Infection	1	1	2	0

#### Secondary malignancies

After a median observation period of 51 months (8 months–148 months), 23 secondary malignancies in 22 patients have been reported, with 13 cases in 12 patients in the RT group (5 leukemias/myelodysplastic syndromes, 1 case each of melanoma, endometrial cancer, Hodgkin lymphoma, cancer of oral cavity, epidermoid cancer of the scalp, thyroid cancer, basal cell carcinoma of the nose, and cancer of unknown primary). The rate of secondary malignancies did not differ significantly between patients with and without RT, respectively (10.1% vs. 7.1%; *p* = 0.504). However, all but one of the leukemias/myelodysplastic syndromes reported occurred in patients who had received both immunochemotherapy and RT. In two patients secondary malignancies occurred in areas which had been irradiated, 1 case of Hodgkin lymphoma and 1 case of thyroid cancer following mediastinal irradiation after an observation time of 31 and 102 months, respectively.

## Discussion

To our knowledge, this is the first detailed report analyzing the role of RT in younger, high-risk patients with aggressive B-cell lymphoma treated on a clinical trial. With a median follow-up of 9.3 years, major short- and long-term toxicities should have been captured. In contrast to other studies, our study stipulated for RT of all patients with bulky disease and/ or involvement of extranodal lesions regardless of the remission status achieved at the end of chemotherapy. Accordingly, 52 patients with CR/CRu after immunochemotherapy did receive local RT. In addition, 50 patients received RT because of an insufficient response (PR, SD) at the end of immunochemotherapy.

As compared to patients without consolidative RT, pre-planned RT resulted in significantly better EFS, PFS, and, most importantly, also OS in patients with bulky disease. Bulky disease has repeatedly been identified as a risk factor for poor survival of DLBCL patients receiving modern immunochemotherapy [19]. In this study, RT not only may have equalized the increased risk for failure but may have contributed to the superior survival of patients given consolidative RT compared to patients not irradiated resulting in the excellent survival of the study population at large. A benefit of RT to extranodal lesions could not be demonstrated. These findings are well in line with a report from our group in older patients (61–80 years) with aggressive B-cell lymphoma [16]. Comparing results from the RICOVER-60 to the RICOVER-noRTh study, which in a comparable study population had abandoned RT, Held et al. reported that older patients with bulky disease significantly benefit from RT while patients with extranodal disease do not. Unfortunately, the relative contribution of consolidative RT to the excellent results of both the RICOVER-60 and the R-MegaCHOEP study remains impossible to quantify because both studies did not randomize patients to RT or no RT.

The DLCL04 trial of the Fondazione Italiana Linfomi (FIL) also investigated dose-intensified treatment approaches for young (18–65 years) high-risk patients (aaIPI: 2–3) with DLBCL or follicular lymphoma grade 3B [20]. In this study, RT was to be administered to isolated FDG-PET-positive areas after immunochemotherapy and, like in our study, to bulky or extranodal disease. Unfortunately, no detailed analysis how many patients received RT for which indication has been reported, letting the question unanswered if RT had a significant impact on the results of this study. Other studies in high-risk patients also compared high-dose therapy and autologous transplantation with standard R-CHOP or variants [21, 22]. The study by Stiff et al. randomized patients after 5 cycles of (R-)CHOP between 3 further cycles of (R-)CHOP and 1 cycle of (R-)CHOP with subsequent autologous transplantation [21]. Except for 12 Gy total body irradiation prior to transplantation, RT was permitted only for biopsy-proven disease residuals or progression. In a further FIL trial, rituximab and high-dose sequential chemotherapy followed by autologous transplantation was tested against R-CHOP [22]. Again, details of RT, which could be administered for bulky (≥5 cm) or residual disease, are not reported.

In contrast to the lack of information how RT influences treatment outcomes in young high-risk patients, a number of studies report on RT as part of the therapeutic concept for early-stage, low-risk [10–13] or older patients [14–16], respectively. The results of these studies cannot directly be compared to our study. However, also these analyses revealed an improvement in EFS with RT for bulky or extranodal disease [10], but no benefit for non-bulky limited-stage DLBCL [11] and uniformly failed to demonstrate an OS improvement. Accordingly, some protocols abandoned RT in favor of intensified chemotherapy-treatment [23, 24].

With the broad application of PET scans for re-staging after immunochemotherapy, the role of RT in patients with risk factors like bulky disease is further challenged. For example, a Canadian retrospective analysis limited RT to PET-positive residues after 6 cycles of R-CHOP demonstrating its efficacy to ameliorate the prognosis for these patients [25]. Prospective studies, however, randomizing patients to RT or no RT for PET-positive lesions at the end of treatment, have not been reported and conclusions regarding the role of RT in patients undergoing re-staging by PET are not yet possible.

Importantly, the potential benefit of RT added to standard immunochemotherapy, has to be carefully weighted against its acute and long-term toxicities. The R-MegaCHOEP trial used doses of 36 Gy, to be administered as involved-field RT, comparable to other analyses [10–12, 14, 25]. Modern trials and guidelines attempt to reduce both RT field size and dose aiming to ameliorate (long-term) toxicity [26–30]. Despite conservative field design and doses, acute toxicities of RT in the R-MegaCHOEP study were mostly mild to moderate. Typical RT-associated toxicities like skin reactions, dysphagia or mucositis, rarely exceeded grade 2. Importantly, RT was not associated with an overall increase in secondary malignancies. There was also no increase in secondary malignancies following RT in the RICOVER-60 [10] and the Italian DLCL04 [20] trial. The FIL investigators described five secondary malignancies (1% of patients) with four solid tumors, none of which was reported to be within a previous RT-field [20]. Although we did not find more secondary malignancies overall, all but one patient developing leukemia or myelodysplastic syndromes had received chemo- and radiotherapy. Long-term follow up of lymphoma patients showed that MDS or acute leukemias occur also in patients not receiving RT [31]. Therefore, the relative contribution of either modality to the occurrence of secondary malignancies remains elusive.

Our study has other limitations: first, patients receiving or not receiving RT as per protocol differed in patient characteristics: the indications for consolidative RT themselves are risk factors of the IPI (extranodal disease) or have repeatedly been described as independent risk factors (bulky disease) [19]. The relatively low number of patients interfered with some subgroup analyses, e.g. regarding the importance of aaIPI. The survival differences between irradiated and not irradiated patients held when patients with protocol violations were excluded from the analysis; however, it remains unknown if patients with an unknown response to immunochemotherapy would have changed the results had their remission status been clear or evaluated by PET. Therefore, the results reported here must be interpreted with caution although our observation that irradiated patients with bulky disease show better EFS, PFS, and OS compared to non-irradiated patients are intriguing and unexpected. This effect persists when considering only patients who completed immunochemotherapy underlining the efficacy of RT. Restricting the analysis to patients with CR/CRu by Cheson criteria after systemic therapy, RT did not result in an improvement of EFS. Unfortunately, these results are difficult to interpret as there was an (expected) imbalance of bulky disease between the irradiated (77%) and non-irradiated patients (31%). Furthermore, with the remission status evaluated by CT scan only, it remains impossible to decide how many of the CR/CRu patients hosted living lymphoma and how many patients had achieved a true PET-negative CR. The R-MegaCHOEP trial was conducted prior to the widespread use of FDG-PET scans in Germany. Current lymphoma trials recommend PET-scans after completion of systemic therapy to decide on further treatment including RT [15, 25]. Further results on RT in such settings are eagerly awaited.

In conclusion, consolidative RT to bulky disease following immunochemotherapy improved survival of young, high-risk patients with aggressive B-cell lymphoma. Adding RT to bulky disease after state-of-the-art systemic therapy for such patients should be strongly considered. Ideally, confirmation of our strategy preferably within a clinical study using modern PET/CT is warranted.

### Supplementary information

Legends supplementary material

Supplementary Table 1

Supplementary Figure 1

## Data Availability

The datasets generated during and/or analyzed during the current study are not publicly available due to limitations by the IRB but are available from the corresponding author on reasonable request.
