# Supplementary material for: Radiotherapy in younger patients with advanced aggressive B-cell lymphoma—long-term results from the phase 3 R-MegaCHOEP trial
Source: Leukemia. 2024 Mar 27;38(5):1099–106. doi: 10.1038/s41375-024-02231-9 (PMC11073960; doi:10.1038/s41375-024-02231-9)
Supplement: Supplementary file 1 — Legends supplementary material [file 41375_2024_2231_MOESM1_ESM.docx]

**Supplementary Figure 1:** Study flow. Doses of the drugs administered with the MegaCHOEP arm (CHOEP with escalated the doses of cyclophosphamide, etoposide, and doxorubicin) varied with each treatment cycle as indicated. Numbers for vincristine and prednisone are indicated as absolute doses. Doses for cyclophosphamide, doxorubicin, and etoposide are given as mg/m^2^. Stars represent one infusion of rituximab. CYC=Cyclophosphamide. DOX=doxorubicin. ETO=etoposide. PRD=prednisone. VCR=vincristine. CHOEP-14=cyclophosphamide, doxorubicin, vincristine, etoposide, prednisone. PBSC=peripheral blood stem cells.

**Supplemenary Table 1:** Remission status after completion of systemic therapy for patients irradiated (RT) or not irradiated (no-RT). CR: complete remission; CRu: unconfirmed complete remission; PD: progressive disease; PR: partial remission; SD: stable disease
