# Supplementary material for: Radiotherapy in younger patients with advanced aggressive B-cell lymphoma—long-term results from the phase 3 R-MegaCHOEP trial
Source: Leukemia. 2024 Mar 27;38(5):1099–106. doi: 10.1038/s41375-024-02231-9 (PMC11073960; doi:10.1038/s41375-024-02231-9)
Supplement: Supplementary file 2 — Supplementary Table 1 [file 41375_2024_2231_MOESM2_ESM.docx]

**Supplementary Table 1:** Remission status after completion of systemic therapy for patients irradiated (RT) or not irradiated (no-RT). CR: complete remission; CRu: unconfirmed complete remission; PD: progressive disease; PR: partial remission; SD: stable disease

| Status after systemic therapy | No-RT | RT |
| --- | --- | --- |
| CR/CRu | 93/141 (66 %) | 52/120 (43 %) |
| PR | 10/141 (7 %) | 48/120 (40 %) |
| SD | 3/141 (2 %) | 2/120 (2 %) |
| PD | 16/141 (11 %) | - |
| unknown | 19/141 (13 %) | 18/120 (15 %) |
