# Supplementary figures and images for: Radiotherapy in younger patients with advanced aggressive B-cell lymphoma—long-term results from the phase 3 R-MegaCHOEP trial
Source: Leukemia. 2024 Mar 27;38(5):1099–106. doi: 10.1038/s41375-024-02231-9 (PMC11073960; doi:10.1038/s41375-024-02231-9)

## Slide 1
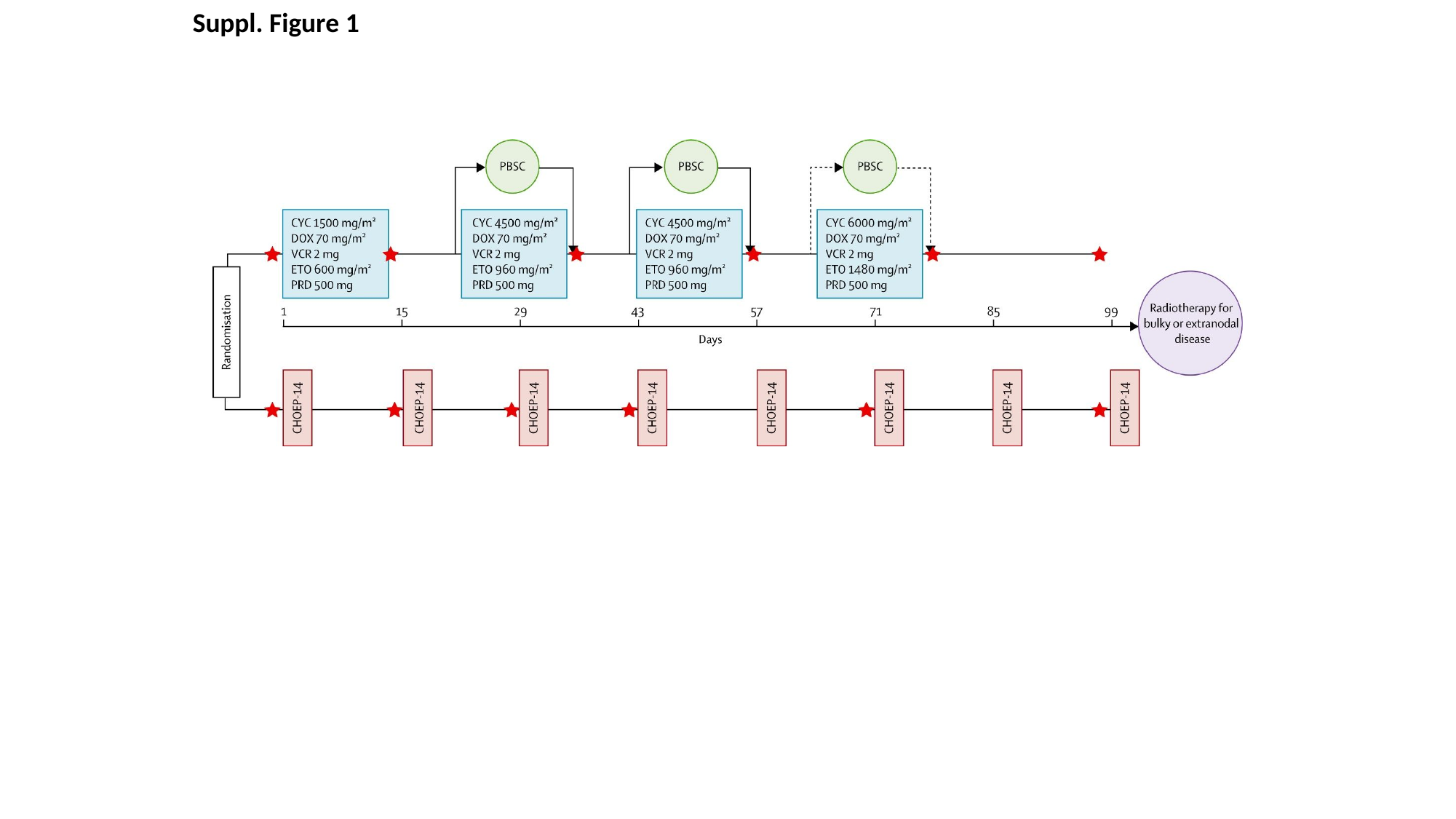

Suppl. Figure 1

Supplement: Supplementary file 3 — Supplementary Figure 1 [file 41375_2024_2231_MOESM3_ESM.pptx]
